# Evaluation of Corneal Sensitivity: Tools We Have

**DOI:** 10.3390/diagnostics15141785

**Published:** 2025-07-15

**Authors:** Ezra Eio, Mingyi Yu, Chang Liu, Isabelle Xin Yu Lee, Regina Kay Ting Wong, Jipson Hon Fai Wong, Yu-Chi Liu

**Affiliations:** 1 Yong Loo Lin School of Medicine, National University of Singapore, Singapore 117597, Singapore; 2 Regenerative Therapy Group, Singapore Eye Research Institute, Singapore 169856, Singapore; 3 Department of Cornea and External Eye Disease, Singapore National Eye Centre, Singapore 169856, Singapore; 4 Ophthalmology and Visual Sciences Academic Clinical Program, Duke-NUS Medical School, Singapore 169857, Singapore

**Keywords:** corneal sensitivity, corneal aesthesiometry, Cochet–Bonnet aesthesiometer, Belmonte non-contact aesthesiometer, Swiss liquid-jet aesthesiometer, Brill aesthesiometer

## Abstract

Corneal sensitivity is an important indicator of corneal health and innervation. Corneal hypoesthesia may be an early indicator of corneal diseases such as neurotrophic keratopathy. Various instruments have been used to measure corneal sensitivity, the first being the Cochet–Bonnet aesthesiometer. Over the years, new devices employing different stimuli have been developed, such as the gas-based Belmonte aesthesiometer, the Swiss liquid-jet aesthesiometer, and the most recently released corneal Brill aesthesiometer. In this review, the progress and advancement of aesthesiometers since their introduction is described. The mechanism, advantages, and disadvantages of these aesthesiometers are discussed and compared. We also report the relationship between corneal sensitivity and corneal innervation in various conditions, including diabetes mellitus, Fuchs’ endothelial dystrophy, dry eye disease, glaucoma, keratoconus, herpes simplex keratitis, post-refractive surgery, and ocular graft-versus-host disease. Through this review, we aim to highlight the importance of the assessment of corneal sensitivity and innervation in the diagnosis, treatment, and monitoring of anterior and posterior segment ocular disorders.

## 1. Introduction

### 1.1. The Cornea, Corneal Sensation, and Neurotrophic Keratopathy

The cornea is the most richly and densely innervated part of the body [1], exceeding even that of the skin by 400 times. It is supplied by a complex network of sensory nerves arising from the ophthalmic branch of the trigeminal nerve, with fibres travelling through the nasociliary nerve and long ciliary nerve, before entering the corneal stroma at the periphery and penetrating the Bowman’s and basal epithelial layer. The corneal nerves serve key roles in the maintenance of tear film, the stimulation of blinking, the perception of foreign bodies and noxious stimuli, and the neurotrophic renewal of the corneal epithelium. These protective and neurotrophic functions of the cornea serve to preserve a healthy ocular surface and maintain epithelial integrity [2].

The overlapping sensory fields of the cornea are supplied by various types of nociceptors which transmit mechanical, pain, and thermal stimuli [3]. Approximately 20% are mechanoreceptors which transmit sharp acute pain via thin myelinated, fast-conducting Aδ-type fibres. The most abundant receptors are the polymodal nociceptors which convey a sharp and sustained pain via unmyelinated, slow-conducting C-type fibres, mediated through chemical substances such as prostaglandins and bradykinin, and heat and mechanical irritation. Lastly, the detection of cold air or fluid is sensed by Aδ and C fibre cold receptors [4].

Any disease, whether ocular or systemic, which causes a lesion along the course of the trigeminal nerve, can lead to corneal neuropathy (Table 1). Various corneal diseases are the most common causes of corneal neuropathy. These include viral or bacterial infection such as herpes simplex virus keratitis and herpes zoster keratoconjunctivitis, as well as chemical burns or physical trauma [1]. Surgical procedures involving the cornea such as various forms of keratoplasty or refractive surgery also cause corneal subbasal nerve plexus transection and, thus, neuropathy [5,6]. Other local ocular etiologies include chronic dry eye disease, chronic contact lens users, keratoconus, and corneal dystrophies [7]. Pharmacological agents such as anti-glaucoma medication [8], intravitreal injections [9], and topical non-steroidal anti-inflammatory agents (NSAIDs) [10] have an adverse effect on corneal sensation. Besides ocular causes, intracranial space-occupying lesions such as a meningioma or neuroma may compress onto the nucleus in the pons or the Gasserian ganglion. Systemic conditions may affect the ophthalmic branch of the trigeminal nerve, the long ciliary nerve, or the nasociliary nerve. These causes include acquired and inherited neuropathies such as diabetic corneal neuropathy, chemotherapy, and Charcot–Marie–Tooth disease [11,12,13]. Autoimmune diseases such as rheumatoid arthritis and fibromyalgia can cause corneal neuropathy [14]. Rarer diseases such as leprosy and metabolic causes including Vitamin A or B12 deficiencies [15,16] have also been reported to result in corneal anaesthesia. Studies have shown that peripheral and, subsequently, central corneal sensitivity decrease in a parabolic fashion with aging [17]. However, the results may differ depending on the aesthesiometer used [18]. Other distinctive patient factors such as iris colour and gender may also impact corneal sensitivity and corneal thresholds to mechanical and chemical stimuli [19].

The loss of protective and neurotrophic sensory innervation leads to corneal epithelial breakdown from repetitive microtraumas, ulceration, and scarring [2]. This condition, known as neurotrophic keratopathy, characterised by repeated epithelial breakdown and poor wound healing, is progressive in nature. The loss of protective blink reflexes and a reduction in tear secretion leads to the impaired perception of noxious stimuli and reduced clearing of the ocular surface, and may eventually lead to the opacification of the cornea and, possibly, blindness [5]. Neurotrophic keratopathy affects 5 or fewer in 10,000 people [20]. It is, therefore, crucial to detect neurotrophic keratopathy early and to monitor disease progression. Using corneal aesthesiometers, early detection can eventually prevent permanent and irreversible corneal damage.

Various aesthesiometers have been used over the years to enable a direct and quantitative assessment of corneal nerve function. In addition to aesthesiometry, a qualitative assessment of corneal nerve morphology, via in vivo confocal microscopy (IVCM), allows for the correlation of corneal sensitivity with corneal innervation [4,21]. In this paper, we aim to discuss the etiologies of corneal neuropathy, the history and types of aesthesiometers, and the advancement of corneal aesthesiometry over the years. We also report the relationship between corneal sensitivity and corneal innervation.

### 1.2. Search Strategy and Selection Criteria

**Figure 1:**
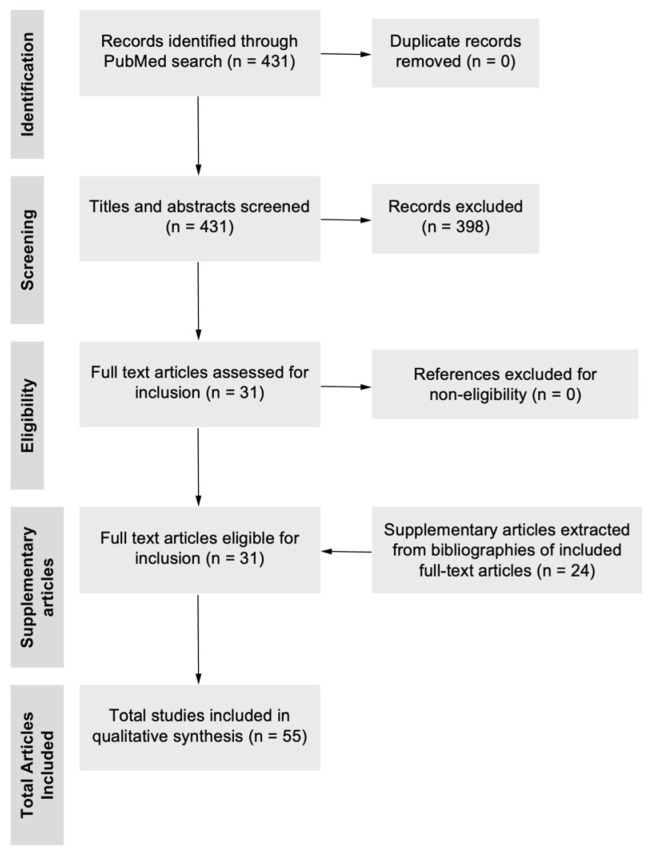
Flow diagram of literature selection process. A search of the database PubMed was performed from the beginning of the study until 24 August 2024 using the search strategy as stated above. After duplicate removal, 431 titles and abstracts were screened, excluding articles based on the inclusion and exclusion criteria, and 31 full-text articles were eligible for inclusion and were surveyed, subsequently adding 24 relevant supplementary articles from currently included full-text articles. A total of 55 articles were used in the writing of the final manuscript.

The authors performed a search on the online database PubMed and Embase for relevant original articles on neurotrophic keratopathy, corneal nerves, and corneal neuropathy. The characteristics of various aesthesiometers, and the relation between corneal sensitivity measured by aesthesiometry, and innervation measured by IVCM were investigated. Articles were included up to July 2024. Two main search strategies were employed, using keywords such as “Corneal sensitivity”, “Corneal sensation”, “Corneal neuropathy”, “Corneal aesthesiometry”, “Cochet-Bonnet aesthesiometer” OR “Swiss Liquid Jet Aesthesiometer” OR “Corneal aesthesiometer Brill”, “In-vivo confocal microscopy”, AND “Corneal aesthesiometry”. Our review only included papers that were written in English as early as 2000 for the earliest original descriptions of corneal structure and neutrotrophic keratopathy. Relevant supplementary articles were extracted from the bibliography of existing articles. Duplicates were removed based on our selection criteria. Out of the original 431 articles derived from the preliminary search strategy, the abstracts were individually screened and further shortlisted. Based on the shortlisted abstracts, the full-text articles were reviewed and 31 were utilised in the final manuscript. The selection process of references is detailed in Figure 1.

## 2. Overview of the History and Types of Aesthesiometers

Before the invention and adoption of aesthesiometry, the cotton wisp test was first used to grossly test corneal sensitivity. The fibres of a cotton tipped applicator would be twirled into a single strand and used to lightly touch the corneal surface, stimulating the blink reflex mediated by the afferent ophthalmic branch of the trigeminal nerve, and the efferent facial nerve [22]. However, this only allowed for a qualitative assessment of corneal sensitivity.

The first quantitative method to evaluate corneal sensitivity, which was later known as aesthesiometry, was first described by Von Frey in 1894 [22]. He attached horse hairs to glass rods, modifying the lengths of hair to vary the pressure exerted on the corneal surface. However, certain factors, such as the condition and age of the hair, air humidity, room temperature, speed, and angle of contact with the surface, affected the measurement. In 1955, Boberg-Ans (University Eye-Clinic, Rigshospitalet, Copenhagen, Denmark) [22] improved on Von Frey’s version by replacing the horse hairs with fine nylon filaments, with a constant diameter but varying lengths. This enabled the adjustment of the rigidity of the filaments when used as a stimulus. In 1966, the Cochet–Bonnet aesthesiometer was introduced, by altering the thread holder from Boberg-Ans’ instrument [23]. The length is inverse to the pressure produced. The Cochet–Bonnet is currently considered the gold standard of corneal sensitivity assessment, being widely used in clinical practice. In the pre-clinical space, the Cochet–Bonnet aesthesiometer has been the predominant instrument of choice, owing to its ease of use, portability, and widely accepted status as a gold standard for assessing mechanosensation. The Cochet–Bonnet aesthesiometer was used to assess the response to mechanical stimuli in mice after the induction of ciliary nerve constriction for neuropathic corneal pain [24]. After the application of the nylon filament, the response was measured by a blink reflex in the mice. In another study involving mice, aimed to showcase the effect of aging on several parameters including corneal sensitivity [25], the Cochet–Bonnet aesthesiometer was similarly used. In mice with diabetic corneal neuropathy, the Cochet–Bonnet aesthesiometer was utilised to show the improved corneal sensitivity after the use of topical and oral peroxisome-proliferator-activated receptor-α agonist [26].

Over the years, the methods and instruments utilised have evolved and advanced significantly. While traditional methods involve direct mechanical stimulation, recent years have seen the development of non-contact aesthesiometers. In 1999, Carlos Belmonte invented the Belmonte Non-contact Corneal Aesthesiometer (NCCA). Instead of direct mechanical stimulation, the NCCA uses a pneumatic stimulus. A calibrated pulse of air of pre-determined pressure and diameter (0.5 mm) is released at a pre-determined distance away from the eye [23]. The air-pulse produces a momentary change in temperature and surface remodelling of the cornea [27], which is commonly perceived by subjects as a “cold” sensitivity. Besides mechanical and thermal stimulation, chemical stimulation was also possible by varying the concentration of the carbon dioxide and air mixture [28]. The CRCERT–Belmonte aesthesiometer is a modified version of the NCCA, incorporating a temperature-controlling device that allows temperature adjustments to eliminate the thermal stimulus [29]. Besides the air-jet stimulus, a new modified aesthesiometer, employing an isotonic saline solution in the form of a liquid jet, was first presented in 2018 [30]. Based on this prototype, the Swiss liquid-jet aesthesiometer for corneal sensitivity (SLACS) was developed [31]. The latest addition to the myriad of options is the new non-contact Brill aesthesiometer, which utilises air pulses but has the added benefit of being portable and mounted on a slit lamp [32], as opposed to older non-contact corneal aesthesiometers which are not handheld.

### 2.1. Cochet–Bonnet Aesthesiometry

**Figure 2:**
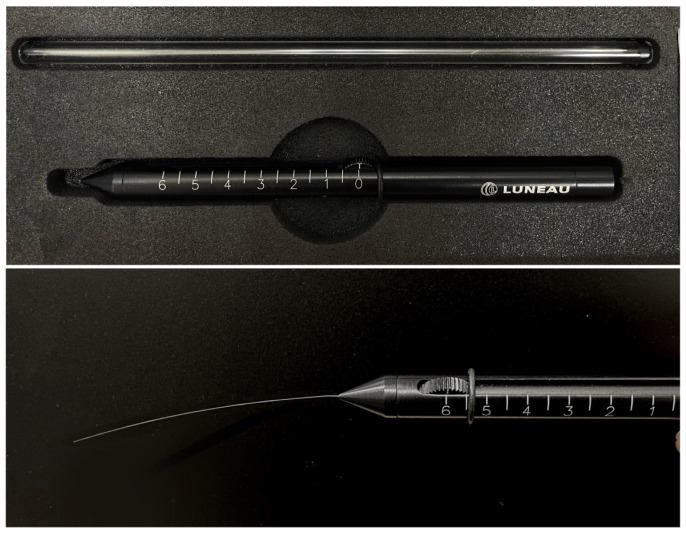
Illustration of the Cochet–Bonnet aesthesiometer used to assess corneal sensitivity. The filament is fully extended to its maximum length (60 mm) and applied to the cornea until the patient reports feeling the physical stimulus.

The Cochet–Bonnet aesthesiometer (Luneau Ophthalmologie; Chartes, France) measures ocular sensitivity by altering the length of the nylon filament, which, in turns, affects the pressure applied to the ocular surface. It is widely considered as the gold standard for aesthesiometry, given its portability and availability via various manufacturers. With two different diameters (0.08 mm and 0.12 mm), the length can be modified from 5 to 60 mm, applying a transmitted pressure of 11 mm/g to 200 mm/g [32]. The thicker filament is used in most studies owing to reduced filament bending and movement, and commercial availability [33]. During the measurement of corneal sensitivity, the longest nylon monofilament is first used. A measurement is counted when the patient feels a mechanical stimulus. If there is no feeling and once a 5° bend is observed [33], the filament length will be gradually decreased until the patient reports a physical sensitivity. A shorter filament will require greater force in order to bend the filament, exerting more pressure on the cornea (Figure 2). The Cochet–Bonnet aesthesiometer is used as the common standard against which new aesthesiometers are compared [27,32], and to evaluate corneal sensitivity in various diseases [34,35,36].

Despite being widely considered as the gold standard for aesthesiometry, the Cochet–Bonnet aesthesiometer also has certain drawbacks, which has led to the development and adoption of new tools to measure corneal sensitivity. The most significant disadvantage is the risk of abrasion of the corneal epithelial surface. When patients are unable to feel the stimulus, the length of the nylon filament will be gradually decreased. The corneal epithelium is fragile and the invasive stimulus, especially at shorter lengths and, thus, higher pressures, may cause epithelial injury [23]. The risk of injury is higher given that many patients undergoing sensory evaluation already have pre-existing corneal disease or surgery. The trauma to the epithelium will further worsen corneal neuropathy by lowering the corneal touch threshold [23].

Besides epithelial injury, there are various user-, patient-, environment-, and instrument-dependent factors that may limit the reliability of the Cochet–Bonnet aesthesiometer. Depending on the clinician who uses the tool, there may be difficulties in standardising the location on the cornea, the angle at which the corneal aesthesiometer is held and at which the cornea is approached, and the measurement of the angle of filament bending. Although a 4% flexure or 5° bend was agreed upon as a common standard, it is impractical to suggest that the operator can accurately measure the angle of filament bending. These will all affect corneal threshold values. From the patients’ perspective, as the Cochet–Bonnet aesthesiometer utilises a contact stimulus, the patient may be hesitant and apprehensive about the visible approaching tool, even flinching and further creating subjectivity [27]. Some studies have chosen to assess only the peripheral cornea to reduce the visual awareness of the instrument. This may limit the number of available corneal locations that can be assessed accurately. Furthermore, studies have reported the effect of ambient humidity on the amount of force exerted by the monofilaments. While keeping the diameter and filament length constant, the force decreases when ambient room humidity levels increase, as there is more absorption of moisture which reduces the material rigidity of the monofilament. The monofilament will thus bend more easily even at the same length [33].

The Cochet–Bonnet aesthesiometer has a limited range of stimulus intensities, and has shown to be less effective in detecting low-intensity stimulus as the minimum possible intensity is often suprathreshold for many patients [37]. In a large majority of subjects, the Cochet–Bonnet aesthesiometer may not be able to measure the true corneal sensitivity threshold. This could be attributed to a more pronounced bend of the filament at its longest lengths (around 60 mm). The limited range will lead to the usage of truncated threshold measurements [27]. When compared to newer aesthesiometers such as the Swiss Liquid or the air-jet non-contact corneal aesthesiometer, these newer instruments were able to detect even lower corneal thresholds as there were corneal responses even at lower stimulus intensities. This would be clinically significant for tracking minute changes and losses of corneal sensitivity over time [18]. Lastly, the Cochet–Bonnet aesthesiometer works by providing a direct mechanical stimulus, but is unable to produce a thermal or chemical stimulus [37]. This may possibly have clinical relevance as different etiologies of neurotrophic keratopathy may impact varying types of corneal fibres which transmit different stimuli. Having the ability to measure different types of nerves may improve the diagnosis, understanding, and, eventually, management of neurotrophic keratopathy.

### 2.2. Gas-Based Non-Contact Corneal Aesthesiometers

The gas-based NCCA operates by generating a controlled jet of air of pre-determined pressure that is directed towards the corneal surface. This air pressure is measured in millibars. The NCCA was developed in an attempt to address some of the drawbacks of the Cochet–Bonnet aesthesiometer. It improves on the sensitivity to lower-intensity stimuli. When the CRCERT–Belmonte (Deriva Global, S.L.; Valencia, Spain), a type of NCCA, and the Cochet–Bonnet aesthesiometer was compared, the corneal sensitivity was measured to be higher with the Belmonte aesthesiometer, as it is able to produce and measure much lower-intensity stimuli and, thus, detect lower corneal sensitivity thresholds, even below the limit of the Cochet–Bonnet aesthesiometer. More than half of the subjects could not be assessed with the standard Cochet–Bonnet aesthesiometer, while, with the Belmonte one, only 11% could not be assessed [38]. This is especially useful at higher sensitivity levels where there may be smaller and more minute changes.

The NCCA is also safe as it is non-invasive and does not possess the risk of epithelial injury. An assessment of the ocular surface after the usage of NCCA showed no evidence of conjunctival hyperemia or epithelial defects on corneal fluorescein staining [39], ensuring the safe assessment of corneal sensitivity recovery post-ocular surgery, or in corneal diseases such as recurrent corneal erosions. The NCCA also measures different stimuli, not only mechanical, but also thermal and chemical, as the mechanism of the air pulse creates a cooling effect when the evaporation of the tear film stimulates the thermoreceptors and, thus, the corneal nerve endings, whereas the carbon dioxide from the gas stimulates chemoreceptors.

However, this creates subjectivity in which stimulus is being measured by the NCCA. While a neural response is undoubtedly recorded, subjects have reported the sensitivity of a general coldness of the eye or a breeze blowing in their eye but were unable to accurately discriminate its location on the cornea [23]. The NCCA may not be measuring the mechanical touch threshold but the threshold to a composite stimulus comprising air pressure and tear evaporation. This would stimulate the Aδ fibres which primarily respond to mechanical stimuli, and the C fibres which respond to thermal stimuli [23]. The inadvertent recruitment of thermal-sensitive nociceptors on top of mechanical receptors may overstimulate and overestimate corneal sensitivity [27]. However, updated versions of the CRCERT–Belmonte aesthesiometer have addressed these limitations by allowing for a controlled temperature to eliminate any thermal component of the stimulus, producing a true mechanical stimulus [31]. Furthermore, the gas jet released by the NCCA is less localised and spread over a wider area as compared to the 0.12 mm filament of the Cochet–Bonnet aesthesiometer. Computational fluid dynamics demonstrates a reduction in the velocity and a pivot in the direction of air flow to a lateral motion upon impact with the central cornea. This exerts pressure on the peripheral region of the cornea one-third that of the apex, limiting the measurement of the corneal sensitivity in specific and precise locations [40].

In terms of portability and convenience, the NCCA loses out to the Cochet–Bonnet aesthesiometer as it has to be mounted onto a slit lamp apparatus which allows for more customisation of the stimulus. The NCCA was briefly commercially available, and would mostly be suitable for use in a dedicated ophthalmology practice and in the academic research setting.

### 2.3. Swiss Liquid-Jet Aesthesiometer for Corneal Sensitivity

In order to overcome some of the deficiencies of the gas-jet aesthesiometer such as the cooling effect and the dispersion of the gas-jet stimulus over a wider area of the cornea, a new prototype was created [30]. This prototype utilised droplets of isotonic saline solution, released from a microvalve of 0.1 mm diameter mounted on a slip lamp. It included a coil for heating and temperature sensor which allows for the precise control of the stimulus temperature to match the temperature of the ocular surface. The temperature regulator eliminates the thermal stimulus as it does not cause secondary evaporative cooling, thus producing a more accurate mechanical stimulus [31]. The stimulus intensity is determined by whether the microvalve is on or off (at a frequency of up to 4 kHz) and the duration (minimally 0.15 ms), while the pressure is kept fixed at 300 mbar [31]. Thus, the stimulus intensity is controlled by the pulse ratio (the ratio of the time when the microvalve is on vs. off). A new modified liquid-jet aesthesiometer, the SLACS (University of Applied Sciences FHNW, Windisch, Switzerland), alters the stimulus intensity by varying the pressure levels from 100 to 1500 mbar with a fixed stimulus duration of 40 ms, providing pulsed stimuli rather than a continuous jet. Compared to the carbon dioxide gas used in the Belmonte aesthesiometer, the pH of the liquid jet may be neutral to eliminate the chemical stimulus, or varied accordingly to create a chemical stimulus. The SLACS can modify pressure levels by increments of 1 mbar, thereby producing a very precise control of the stimulus intensity, and is thus able to detect even subtler changes in corneal sensitivity. A high-speed recording of the liquid jet making contact with the ocular surface showed the stimulus reaches a precise and localised region of the cornea, with little vertical and horizontal displacement [31].

Despite the improvements in the precise localisation of the stimulus, there still exists some uncertainty in the exact pressure exerted on the cornea, as this is affected by the speed and force of the release jet. Although it improves on the NCCA, the measurements may not be as precise as those of the Cochet–Bonnet aesthesiometer. The psychophysical stimulus makes it difficult to determine exactly which stimulus is being measured—the mechanical, thermal, or chemical. Being a relatively newer device like the gas-based NCCA, the SLACS is not widely available commercially and mainly used within research settings, not sharing the convenience, portability, and accessibility of the Cochet–Bonnet aesthesiometer.

### 2.4. Non-Contact Brill Aesthesiometer

**Figure 3:**
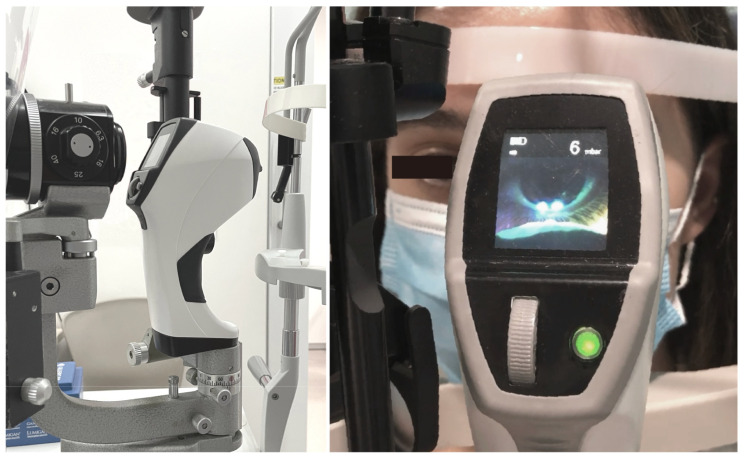
Setup of the Brill aesthesiometer used as a slip lamp attachment. The Brill aesthesiometer features a camera and small screen to accurately target the area for corneal stimulation, using the alignment of two converging LED lights projected onto the cornea to ensure precise stimulation at the appropriate distance.

The new corneal Brill aesthesiometer (Brill Engines, S.L; Barcelona, Spain) was introduced in 2023 and is a non-contact, gas-based aesthesiometer like the Belmonte one. The device uses ambient air instead of gas emitted from cylinders. The air is released from an outlet nozzle placed 4 mm from the corneal surface. At this distance, two light-emitting diode (LED) lights converge on the subject’s corneal surface. A screen and camera allow the operator to adjust and pinpoint the exact area for stimulation at a correct distance [32]. It can deliver pulses of air at five different levels of intensities and a pressure range of 2–10 mbar [41]. The Brill aesthesiometer can be used as a handheld device or mounted on a slit lamp (Figure 3), matching the Cochet–Bonnet aesthesiometer in portability and accessibility. It is non-invasive and does not risk causing epithelial injury and trauma. Although the Brill aesthesiometer is a relatively newer aesthesiometer and not many studies have been performed on it, current results show that the Brill was effective in evaluating corneal sensitivity in both healthy individuals and patients with ocular diseases such as dry eye disease and ocular graft-versus-host disease, demonstrating good repeatability [32,42,43]. Corneal sensitivity obtained using the Brill aesthesiometer showed a significant positive correlation and a good agreement with that obtained from the Cochet–Bonnet aesthesiometer [32,42,43]. The most recent and largest study thus far examining the repeatability and reproducibility of the Brill aesthesiometer by Ruiz-Lozano [42] showed excellent intra- and inter-observed repeatability (ICC > 0.9). However, the values from the Brill and Cochet–Bonnet aesthesiometers are not interchangeable, with a significant difference between the mean corneal sensitivity thresholds from both devices: 0.052 ± 0.021 mN for Brill vs. 0.046 ± 0.005 mN, *p* = 0.001 for Cochet.

However, the Brill aesthesiometer shares similar limitations to the Belmonte one, being an air-based aesthesiometer. The gas jet produces a mechanical, thermal, and chemical stimulus that all have a shared mechanism in stimulating the corneal surface, due to evaporative cooling and tear film depletion. The gas jet is dispersed across a wider area on the ocular surface, spreading over a diameter of 0.5 mm, which may compromise the precise localisation of the stimulus on the cornea. As there are only five intensity levels, with the pressure of the air stimulus ranging from 2.0 mbar to 10.0 mbar, the Brill aesthesiometer may not be able to detect smaller changes in corneal sensitivity. In a recent study, there were subjects that could not feel the stimulus at the maximum pressure (10 mbar) and, hence, the measurement had to be taken as 11 mbar, one above the maximum value [41]. More extensive study on the reliability and accuracy of the Brill aesthesiometer will be helpful for guiding its future use for the management of patients in clinical settings and in research.

Table 2 summarises the main characteristics of different types of aesthesiometers.

## 3. Correlation Between Corneal Sensitivity and Innervation

The quantitative relationship between corneal sensitivity and innervation has been explored and studied. As stated in the previous section, studies ranging across different diseases show a correlation between various parameters, including the subbasal nerve density, number, length, branching and tortuosity, with decreased corneal sensitivity. These diseases include dry eye disease [44], glaucoma [45], keratoconus [46], herpes keratitis [47], post-refractive surgery [48], diabetes [49], and Fuchs’ corneal dystrophy [50]. In type 1 diabetes, corneal sensitivity was significantly and positively correlated with the number of long nerve fibre bundles (r = 0.42; *p* = 0.048) [49]. IVCM shows a loss of nerve fibre bundles which precedes sensitivity impairment, with mild–moderate neuropathy showing decreased corneal nerve innervation but only severe neuropathy presenting with reduced sensitivity [49]. Similar findings were reported by Takavoli et al., who described a significant reduction in corneal sensitivity assessed using the Cochet–Bonnet aesthesiometer in mild, moderate, and severe diabetic peripheral neuropathy, while NCCA measurements showed a significant reduction in moderate and severe cases (all *p* < 0.05), with neuropathy severity evaluated using the Neuropathy Deficit Score [51].

For patients with Fuchs’ endothelial dystrophy, Aggarwal et al. reported that corneal sensitivity assessed by the Cochet-Bonnet aesthesiometer was significantly correlated with the number (r = 0.47; *p* = 0.010) and length (r = 0.32; *p* = 0.044) of subbasal nerves [50]. Another study on Fuchs’ endothelial dystrophy by Dikmetas et al. demonstrated Cochet–Bonnet measurements were significantly correlated with corneal subbasal nerve plexus densities (r = 0.46, *p* = 0.025) and nerve tortuosity (r = 0.44, *p* = 0.031) [52].

With regard to dry eye disease, Labbé et al. found a significant positive correlation between corneal sensitivity, as measured by the Cochet–Bonnet aesthesiometer, and both the density (r = 0.64; *p* = 0.045) and number of subbasal nerves (r = 0.65; *p* = 0.043) [53]. Benítez-del-Castillo et al. found that subbasal nerve density correlated significantly with mechanical, chemical, and thermal sensitivity thresholds assessed by NCCA (r = −0.79, r = −0.80, and r = −0.63, respectively; all *p* < 0.001) [44].

When looking at the correlation between corneal sensitivity and corneal nerve tortuosity, two studies which performed analyses on glaucoma patients revealed an inverse relationship between Cochet–Bonnet measurements and subbasal nerve tortuosity (r = −0.54 and r = −0.62, both *p* < 0.05) [45,53]. This inverse relationship indicates a worse corneal nerve morphology correlating with corneal sensitivity.

In keratoconus, the corneal sensitivity assessed by NCCA significantly correlated to subbasal nerve density (r = −0.31, *p* = 0.001) [46]. Additionally, a study on herpes simplex keratitis found that Cochet–Bonnet measurements were significantly correlated to subbasal nerve density (r = 0.65, *p* < 0.0001) and main nerve trunks (r = 0.62, *p* < 0.0002) [47]. In patients who have undergone refractive surgery, the corneal sensitivity assessed by the Cochet–Bonnet aesthesiometer was significantly correlated with subbasal corneal nerve fibre bundle density in both post-laser-assisted in situ keratomileusis (LASIK) (r = 0.93, *p* < 0.001) and post-laser epithelial keratomileusis (LASEK) eyes (r = 0.68, *p* < 0.001) [54].

Recently, Surico et al. reported a significant negative correlation between the corneal sensitivity assessed by the Brill aesthesiometer and the total (r = −0.83; *p* < 0.001), main trunk (r = −0.62; *p* < 0.001), and branch (r = −0.72; *p* < 0.001) nerve densities in patients with ocular graft-versus-host disease [43]. These results suggest that corneal nerves’ quantity and quality can be directly correlated with corneal sensitivity assessment using aesthesiometry. Table 3 summarises the key findings of the literature on the correlation between corneal sensitivity and subbasal nerve plexus parameters.

However, there are some studies which have shown no apparent correlation between corneal sensitivity and corneal nerve metrics. This can be explained as follows:

Firstly, at an early stage of the disease with low severity, there may not have been a drop in corneal nerve metrics and innervation. In the study on diabetic peripheral neuropathy by Rosenberg et al., the early stages of the disease with mild–moderate peripheral neuropathy reveals decreased corneal innervation, but not reduced corneal sensitivity. Only patients with severe peripheral neuropathy presents with corneal neuropathy [49,51]. In Fuchs’ endothelial dystrophy, there may be certain parts of the subbasal nerve plexus which undergo alteration and are rendered dysfunctional, for example, the central cornea, before finally leading to the loss of the subbasal nerve plexus in the entire cornea [52,55].

Secondly, the current instruments and methods available for both the measurement of corneal sensitivity in aesthesiometry and corneal morphology on the IVCM evaluation may not be adequately sensitive enough to detect minute and early changes. For IVCM, improvements can be made in increasing the field of view and automatic quantification [53,56]. The small field of view in IVCM (0.16 mm^2^) limits the comprehensive overview of the entire cornea. Improvements in the field of view and reconstruction will enhance the corneal morphology assessment [57]. In addition, there have been new software developed which enable the precise calculation and measurement of subbasal nerve metrics [58]. For corneal aesthesiometry, one drawback is that contact aesthesiometry merely evaluates mechanoreceptors while missing out on chemoreceptors and thermoreceptors, thus not being an absolute and all-encompassing measure of corneal sensitivity [52]. Furthermore, many of the studies examining the correlation between corneal sensitivity and corneal innervation use contact aesthesiometry (Cochet–Bonnet) [47,49,50,53], while other studies used non-contact aesthesiometry [44,46]. As these readings are not interchangeable, standardising the use of aesthesiometers may help to elucidate a clearer correlation between corneal sensitivity and innervation.

Thirdly, as of now, IVCM most reliably and sensitively detects and images subbasal nerve bundles. However, epithelial and stromal nerves, due to their far lower density, are sporadically missed out by IVCM [59]. Hence, functional neural loss that occurs in the epithelial or stromal nerves, or even in the synaptic or nuclear level, may not be noticeable on IVCM [60].

## 4. Conclusions and Future Directions

The ideal next-generation aesthesiometer should integrate multiple types of stimuli, such as mechanical, thermal, and chemical stimuli, in a controlled and reproducible manner, while also balancing precision, accuracy, accessibility, and ease of use in clinical settings. A gas-based system remains promising due to its non-contact nature and safety profile. Besides the diagnosis of neuropathic corneas, aesthesiometers also plays a role in monitoring the disease progression and the status of corneal reinnervation after treatment. Thus, the assessment of the structure and function of the corneal nerves continues to be a highly essential and beneficial clinical priority.

## Figures and Tables

**Table 1:** Etiologies of corneal neuropathy.

Etiology of Corneal Neuropathy	
Ocular	Herpetic, viral or bacterial infections Chemical burns, physical injuries Corneal surgery Corneal dystrophies Limbal stem cell deficiency Keratoconus Long-term contact lens use Pharmacological agents (anti-glaucoma eyedrops, intravitreal injections, topical NSAIDs)
Systemic	Acquired neuropathy: diabetic corneal neuropathy, chemotherapy-induced Inherited neuropathies: Charcot-Marie-Tooth Autoimmune: rheumatoid arthritis, fibromyalgia Metabolic: vitamin A deficiency, vitamin B12 deficiency
Intracranial	Neoplasm: meningioma, neuroma Aneurysm Stroke Neurosurgery, surgical injury to the trigeminal nerve
Genetic	Riley-Day syndrome Goldenhar syndrome Familial corneal neuropathy

**Table 2:** Characteristics of different aesthesiometers.

Instrument	Mechanism/Principle	Advantages	Disadvantages
Cochet–Bonnet aesthesiometer	Mechanical stimulus using a nylon filament with modifiable length (5 to 60 mm), being able to exert a pressure of 11 mm/g to 200 mm/g	Commonly used in clinical practice and research as the current gold standard of aesthesiometry; Commercially available; Portable.	Risk of corneal epithelial injury; Subjective operator-dependent factors: location on cornea, angle, and filament bending Environmental, and instrument-dependent factors: ambient humidity; Limited range of stimulus intensities.
Gas-based non-contact corneal aesthesiometer	Generating a controlled jet of air directed towards the corneal surface which exerts mechanical pressure on the surface while producing a cooling effect on the cornea	Non-invasive, and no risk of epithelial injury; Higher sensitivity with lower-intensity stimuli as compared to Cochet–Bonnet; Produces mechanical, chemical, and thermal stimuli.	Subjectivity in the type of stimuli being measured; Gas stimulus is dispersed, which limits the precise measurement of corneal sensitivity to a specific location; Complex and less accessible, requiring slit lamp mounting.
Swiss liquid-jet aesthesiometer	Liquid jet with an isotonic saline solution released from a microvalve mounted on a slit lamp with a temperature sensor	Temperature regulator handles thermal stimuli; Neutral isotonic solution removes chemical stimuli; Precise control of pulsed jet stimulus in 1 mbar increments; Well-localised area stimulated on cornea.	Less precise area of stimuli compared to Cochet–Bonnet aesthesiometer; Complex and less accessible, requiring slit lamp mounting; Not commercially available.
Brill aesthesiometer	Ambient air pulse emitted from an outlet nozzle placed 4 mm away from the corneal surface	Non-invasive, and no risk of epithelial injury; Versatile, used as a handheld device or can be mounted on a slit lamp; Aid of two LED lights for localisation.	Limited range of stimulus intensities (5 levels, from 2 to 10 mbar); Gas stimulus less-localised than Cochet–Bonnet aesthesiometer; Not extensively researched and studied yet.

**Table 3:** Corneal sensitivity correlated with subbasal nerve plexus parameters measured on IVCM in various ocular conditions.

Diseases	Studies	Study Design	Aesthesiometer	Study Findings
Type 1 diabetes	Rosenberg et al. [49]	Cross-sectional study; *n* = 23	Cochet–Bonnet aesthesiometer	Corneal sensitivity significantly and positively correlated with the number of long nerve fibre bundles (*r* = 0.42; *p* = 0.048). Nerve fibre loss precedes corneal sensitivity impairment, with reduced sensitivity only observed in cases of severe neuropathy.
Type 1/type 2 diabetes	Tavakoli et al. [51]	Cross-sectional controlled study; *n* = 165	Cochet–Bonnet aesthesiometer and non-contact corneal aesthesiometer	Corneal sensitivity assessed with the Cochet–Bonnet aesthesiometer was significantly reduced in mild, moderate, and severe diabetic peripheral neuropathy. Corneal sensitivity assessed with the non-contact corneal aesthesiometer showed a significant reduction in moderate and severe cases of diabetic peripheral neuropathy.
Fuchs’ endothelial dystrophy	Aggarwal et al. [50]	Prospective, cross-sectional, controlled study; *n* = 30	Cochet–Bonnet aesthesiometer	Corneal sensitivity significantly correlated with the number (*r* = 0.47; *p* = 0.010) and length (*r* = 0.32; *p* = 0.044) of subbasal corneal nerves.
	Dikmetas et al. [52]	Retrospective, cross-sectional study; *n* = 49	Cochet–Bonnet aesthesiometer	Corneal sensitivity significantly correlated with corneal subbasal nerve plexus densities (*r* = 0.46, *p* = 0.025) and nerve tortuosity (*r* = 0.44, *p* = 0.031).
Dry eye disease	Labbé et al. [53]	Cross-sectional study; *n* = 22	Cochet–Bonnet aesthesiometer	Corneal sensitivity significantly positively correlated with both the density (*r* = 0.64; *p* = 0.045) and number of subbasal nerves (*r* = 0.65; *p* = 0.043).
	Benítez-Del-Castillo et al. [44]	Prospective, cross-sectional, controlled study; *n* = 41	Non-contact corneal aesthesiometer	Subbasal nerve density correlated significantly with mechanical, chemical, and thermal sensitivity thresholds (*r* = −0.79, *r* = −0.80, and *r* = −0.63, respectively; all *p* < 0.001).
Glaucoma	Martone et al. [45]	Comparative retrospective study; *n* = 104	Cochet–Bonnet aesthesiometer	Significant correlation was observed between corneal sensitivity and nerve tortuosity (*r* = −0.54, *p* < 0.05).
	Labbé et al. [53]	Cross-sectional study; *n* = 24	Cochet–Bonnet aesthesiometer	Corneal sensitivity correlated negatively with the tortuosity of the subbasal nerves (*r* = 0.623; *p* = 0.03).
Keratoconus	Patel et al. [46]	Cross-sectional study; *n* = 58	Non-contact corneal aesthesiometer	Corneal sensitivity significantly correlated to subbasal nerve density (*r* = −0.31, *p* = 0.001).
Herpes simplex keratitis	Hamrah et al. [47]	Prospective, cross-sectional, controlled study; *n* = 46	Cochet–Bonnet aesthesiometer	Corneal sensitivity was significantly correlated to subbasal nerve density (*r* = 0.65, *p* < 0.0001) and main nerve trunks (*r* = 0.62, *p* < 0.0002).
Post-refractive surgery	Lee et al. [54]	Prospective, nonrandomised comparative clinical trial; *n* = 54	Cochet–Bonnet aesthesiometer	Corneal sensitivity was significantly correlated with subbasal corneal nerve fibre bundle density in both post-laser-assisted in situ keratomileusis (LASIK) (*r* = 0.93, *p* < 0.001) and post-laser epithelial keratomileusis (LASEK) eyes (*r* = 0.68, *p* < 0.001).
Ocular graft-versus-host disease	Surico et al. [43]	Retrospective study; *n* = 36	Brill aesthesiometer	Significant negative correlations were observed between corneal sensitivity and the total (*r* = −0.83; *p* < 0.001), main trunk (*r* = −0.62; *p* < 0.001), and branch (*r* = −0.72; *p* < 0.001) nerve densities.

## Data Availability

The original contributions presented in this study are included in this article material; further inquiries can be directed to the corresponding author.

## Abbreviations

The following abbreviations are used in this manuscript:

NSAIDs	Non-steroidal anti-inflammatory agents
IVCM	In vivo confocal microscopy
NCCA	Non-contact corneal aesthesiometer
SLACS	Swiss liquid-jet aesthesiometer for corneal sensitivity
LED	Light-emitting diode
LASIK	Laser-assisted in situ keratomileusis
LASEK	Laser-assisted epithelial keratomileusis

## References

[1] Bonini S., Rama P., Olzi D., Lambiase A. (2003). Neurotrophic keratitis. Eye.

[2] Feinberg K., Tajdaran K., Mirmoeini K., Daeschler S.C., Henriquez M.A., Stevens K.E., Mulenga C.M., Hussain A., Hamrah P., Ali A. (2023). The Role of Sensory Innervation in Homeostatic and Injury-Induced Corneal Epithelial Renewal. Int. J. Mol. Sci..

[3] Crabtree J.R., Tannir S., Tran K., Boente C.S., Ali A., Borschel G.H. (2024). Corneal Nerve Assessment by Aesthesiometry: History, Advancements, and Future Directions. Vision.

[4] Sacchetti M., Lambiase A. (2014). Diagnosis and management of neurotrophic keratitis. Clin. Ophthalmol..

[5] Yang A.Y., Chow J., Liu J. (2018). Corneal Innervation and Sensation: The Eye and Beyond. Yale J. Biol. Med..

[6] Liu Y.C., Jung A.S.J., Chin J.Y., Yang L.W.Y., Mehta J.S. (2020). Cross-sectional Study on Corneal Denervation in Contralateral Eyes Following SMILE Versus LASIK. J. Refract. Surg..

[7] Liu C., Lin M.T., Lee I.X.Y., Wong J.H.F., Lu D., Lam T.C., Zhou L., Mehta J.S., Ong H.S., Ang M. (2024). Neuropathic Corneal Pain: Tear Proteomic and Neuromediator Profiles, Imaging Features, and Clinical Manifestations. Am. J. Ophthalmol..

[8] Romero-Díaz de León L., Morales-León J.E., Ledesma-Gil J., Navas A. (2016). Conjunctival and corneal sensitivity in patients under topical antiglaucoma treatment. Int. Ophthalmol..

[9] Polat O.A., Şener H., Erkiliç K. (2022). Corneal Nerve Fiber and Sensitivity Loss After Repeated Intravitreal Anti-VEGF Injections: An In Vivo Confocal Microscopy Study. Cornea.

[10] Singer D.D., Kennedy J., Wittpenn J.R. (2015). Topical NSAIDs effect on corneal sensitivity. Cornea.

[11] Chiang J.C.B., Goldstein D., Trinh T., Au K., Mizrahi D., Muhlmann M., Crowe P., O’Neill S., Edwards K., Park S.B. (2021). A Cross-Sectional Study of Sub-Basal Corneal Nerve Reduction Following Neurotoxic Chemotherapy. Transl. Vis. Sci. Technol..

[12] Mansoor H., Tan H.C., Lin M.T., Mehta J.S., Liu Y.C. (2020). Diabetic Corneal Neuropathy. J. Clin. Med..

[13] So W.Z., Qi Wong N.S., Tan H.C., Yu Lin M.T., Yu Lee I.X., Mehta J.S., Liu Y.C. (2022). Diabetic corneal neuropathy as a surrogate marker for diabetic peripheral neuropathy. Neural Regen. Res..

[14] Kim I.G., Lee J.H., Kim S.S. (2012). Reduced corneal sensitivity in patients with rheumatoid arthritis. Cornea.

[15] Rocha E.M., Gutierrez D.R. (2024). Hypovitaminosis A: A hidden cause of neurotrophic keratitis. Arq. Bras. Oftalmol..

[16] Nassiri N., Assarzadegan F., Shahriari M., Norouzi H., Kavousnezhad S., Nassiri N., Sheibani K. (2018). Vitamin B12 Deficiency as a Cause of Neurotrophic Keratopathy. Open Ophthalmol. J..

[17] Roszkowska A.M., Colosi P., Ferreri F.M., Galasso S. (2004). Age-related modifications of corneal sensitivity. Ophthalmologica.

[18] Nosch D.S., Käser E., Bracher T., Joos R.E. (2023). Age-Related Changes in Corneal Sensitivity. Cornea.

[19] Acosta M.C., Alfaro M.L., Borrás F., Belmonte C., Gallar J. (2006). Influence of age, gender and iris color on mechanical and chemical sensitivity of the cornea and conjunctiva. Exp. Eye Res..

[20] Dua H.S., Said D.G., Messmer E.M., Rolando M., Benitez-Del-Castillo J.M., Hossain P.N., Shortt A.J., Geerling G., Nubile M., Figueiredo F.C. (2018). Neurotrophic keratopathy. Prog. Retin. Eye Res..

[21] Liu Y.C., Lin M.T., Mehta J.S. (2021). Analysis of corneal nerve plexus in corneal confocal microscopy images. Neural Regen. Res..

[22] Draeger J., Draeger J. (1984). Development of the Various Methods of Esthesiometry. Corneal Sensitivity: Measurement and Clinical Importance.

[23] Murphy P.J., Patel S., Marshall J. (1996). A new non-contact corneal aesthesiometer (NCCA). Ophthalmic Physiol. Opt..

[24] Seyed-Razavi Y., Kenyon B.M., Qiu F., Harris D.L., Hamrah P. (2023). A novel animal model of neuropathic corneal pain-the ciliary nerve constriction model. Front. Neurosci..

[25] De Silva M.E.H., Hill L.J., Downie L.E., Chinnery H.R. (2019). The Effects of Aging on Corneal and Ocular Surface Homeostasis in Mice. Investig. Ophthalmol. Vis. Sci..

[26] Mansoor H., Lee I.X.Y., Lin M.T.-Y., Ang H.P., Xue Y.C., Krishaa L., Patil M., Koh S.-K., Tan H.C., Zhou L. (2024). Topical and oral peroxisome proliferator-activated receptor-α agonist ameliorates diabetic corneal neuropathy. Sci. Rep..

[27] Nosch D.S., Käser E., Bracher T., Joos R.E. (2024). Clinical application of the Swiss Liquid Jet Aesthesiometer for corneal sensitivity measurement. Clin. Exp. Optom..

[28] Belmonte C., Acosta M.C., Schmelz M., Gallar J. (1999). Measurement of corneal sensitivity to mechanical and chemical stimulation with a CO2 esthesiometer. Investig. Ophthalmol. Vis. Sci..

[29] Stapleton F., Tan M.E., Papas E.B., Ehrmann K., Golebiowski B., Vega J., Holden B.A. (2004). Corneal and conjunctival sensitivity to air stimuli. Br. J. Ophthalmol..

[30] Ehrmann K., Saha M., Falk D. (2018). A novel method to stimulate mechanoreceptors and quantify their threshold values. Biomed. Phys. Eng. Express.

[31] Nosch D.S., Oscity M., Steigmeier P., Käser E., Loepfe M., Joos R.E. (2022). Working principle and relevant physical properties of the Swiss Liquid Jet Aesthesiometer for Corneal Sensitivity (SLACS) evaluation. Ophthalmic Physiol. Opt..

[32] Merayo-Lloves J., Gómez Martín C., Lozano-Sanroma J., Renedo Laguna C. (2024). Assessment and safety of the new esthesiometer BRILL: Comparison with the Cochet-Bonnet Esthesiometer. Eur. J. Ophthalmol..

[33] Lum E., Murphy P.J. (2018). Effects of ambient humidity on the Cochet-Bonnet aesthesiometer. Eye.

[34] Reinstein D.Z., Archer T.J., Gobbe M., Bartoli E. (2015). Corneal sensitivity after small-incision lenticule extraction and laser in situ keratomileusis. J. Cataract. Refract. Surg..

[35] Bucher F., Adler W., Lehmann H.C., Hos D., Steven P., Cursiefen C., Heindl L.M. (2014). Corneal nerve alterations in different stages of Fuchs’ endothelial corneal dystrophy: An in vivo confocal microscopy study. Graefes Arch. Clin. Exp. Ophthalmol..

[36] Cruzat A., Hamrah P., Cavalcanti B.M., Zheng L., Colby K., Pavan-Langston D. (2016). Corneal Reinnervation and Sensation Recovery in Patients With Herpes Zoster Ophthalmicus: An In Vivo and Ex Vivo Study of Corneal Nerves. Cornea.

[37] Murphy P.J., Lawrenson J.G., Patel S., Marshall J. (1998). Reliability of the non-contact corneal aesthesiometer and its comparison with the Cochet-Bonnet aesthesiometer. Ophthalmic Physiol. Opt..

[38] Golebiowski B., Papas E., Stapleton F. (2011). Assessing the sensory function of the ocular surface: Implications of use of a non-contact air jet aesthesiometer versus the Cochet-Bonnet aesthesiometer. Exp. Eye Res..

[39] Tesón M., Calonge M., Fernández I., Stern M.E., González-García M.J. (2012). Characterization by Belmonte’s gas esthesiometer of mechanical, chemical, and thermal corneal sensitivity thresholds in a normal population. Investig. Ophthalmol. Vis. Sci..

[40] Golebiowski B., Lim M., Papas E., Stapleton F. (2013). Understanding the stimulus of an air-jet aesthesiometer: Computerised modelling and subjective interpretation. Ophthalmic Physiol. Opt..

[41] Villalba M., Sabates V., Orgul S., Perez V.L., Swaminathan S.S., Sabater A.L. (2024). Detection of Subclinical Neurotrophic Keratopathy by Noncontact Esthesiometry. Ophthalmol. Ther..

[42] Ruiz-Lozano R.E., Quiroga-Garza M.E., Ramos-DÁVila E.M., PantaleÓN-GarcÍA J., Khodor A.L.I., Komai S., Rodriguez-Gutierrez L.A., Ma S., Mousa H.M., Mattes R. (2025). Comparative Evaluation of the Corneal Sensitivity Thresholds between the Novel Non-Contact and Cochet-Bonnet Esthesiometers. Am. J. Ophthalmol..

[43] Surico P.L., Saricay L.Y., Singh R.B., Kahale F., Romano F., Dana R. (2022). Corneal Sensitivity and Neuropathy in Patients With Ocular Graft-Versus-Host Disease. Cornea.

[44] Benítez-Del-Castillo J.M., Acosta M.C., Wassfi M.A., Díaz-Valle D., Gegúndez J.A., Fernandez C., García-Sánchez J. (2007). Relation between corneal innervation with confocal microscopy and corneal sensitivity with noncontact esthesiometry in patients with dry eye. Investig. Ophthalmol. Vis. Sci..

[45] Martone G., Frezzotti P., Tosi G.M., Traversi C., Mittica V., Malandrini A., Pichierri P., Balestrazzi A., Motolese P.A., Motolese I. (2009). An in vivo confocal microscopy analysis of effects of topical antiglaucoma therapy with preservative on corneal innervation and morphology. Am. J. Ophthalmol..

[46] Patel D.V., Ku J.Y., Johnson R., McGhee C.N. (2009). Laser scanning in vivo confocal microscopy and quantitative aesthesiometry reveal decreased corneal innervation and sensation in keratoconus. Eye.

[47] Hamrah P., Cruzat A., Dastjerdi M.H., Zheng L., Shahatit B.M., Bayhan H.A., Dana R., Pavan-Langston D. (2010). Corneal sensation and subbasal nerve alterations in patients with herpes simplex keratitis: An in vivo confocal microscopy study. Ophthalmology.

[48] Stachs O., Zhivov A., Kraak R., Hovakimyan M., Wree A., Guthoff R. (2010). Structural-functional correlations of corneal innervation after LASIK and penetrating keratoplasty. J. Refract. Surg..

[49] Rosenberg M.E., Tervo T.M., Immonen I.J., Müller L.J., Grönhagen-Riska C., Vesaluoma M.H. (2000). Corneal structure and sensitivity in type 1 diabetes mellitus. Investig. Ophthalmol. Vis. Sci..

[50] Aggarwal S., Cavalcanti B.M., Regali L., Cruzat A., Trinidad M., Williams C., Jurkunas U.V., Hamrah P. (2018). In Vivo Confocal Microscopy Shows Alterations in Nerve Density and Dendritiform Cell Density in Fuchs’ Endothelial Corneal Dystrophy. Am. J. Ophthalmol..

[51] Tavakoli M., Kallinikos P.A., Efron N., Boulton A.J., Malik R.A. (2007). Corneal sensitivity is reduced and relates to the severity of neuropathy in patients with diabetes. Diabetes Care.

[52] Dikmetas O., Kocabeyoglu S., Mocan M.C., Karahan S., İrkec M. (2021). The relationship between corneal subbasal nerve density and corneal sensitivity in patients with Fuchs endothelial corneal dystrophy. Indian J. Ophthalmol..

[53] Labbé A., Alalwani H., Van Went C., Brasnu E., Georgescu D., Baudouin C. (2012). The relationship between subbasal nerve morphology and corneal sensation in ocular surface disease. Investig. Ophthalmol. Vis. Sci..

[54] Lee S.J., Kim J.K., Seo K.Y., Kim E.K., Lee H.K. (2006). Comparison of corneal nerve regeneration and sensitivity between LASIK and laser epithelial keratomileusis (LASEK). Am. J. Ophthalmol..

[55] Han S.B., Liu Y.C., Liu C., Mehta J.S. (2024). Applications of Imaging Technologies in Fuchs Endothelial Corneal Dystrophy: A Narrative Literature Review. Bioengineering.

[56] Chin J.Y., Yang L.W.Y., Ji A.J.S., Nubile M., Mastropasqua L., Allen J.C., Mehta J.S., Liu Y.C. (2020). Validation of the Use of Automated and Manual Quantitative Analysis of Corneal Nerve Plexus Following Refractive Surgery. Diagnostics.

[57] Stewart S., Liu Y.C., Lin M.T., Mehta J.S. (2021). Clinical Applications of In Vivo Confocal Microscopy in Keratorefractive Surgery. J. Refract. Surg..

[58] Patel D.V., Tavakoli M., Craig J.P., Efron N., McGhee C.N. (2009). Corneal sensitivity and slit scanning in vivo confocal microscopy of the subbasal nerve plexus of the normal central and peripheral human cornea. Cornea.

[59] Müller L.J., Marfurt C.F., Kruse F., Tervo T.M. (2003). Corneal nerves: Structure, contents and function. Exp. Eye Res..

[60] Erie J.C., McLaren J.W., Hodge D.O., Bourne W.M. (2005). The effect of age on the corneal subbasal nerve plexus. Cornea.

